# PI3-Kinase γ Promotes Rap1a-Mediated Activation of Myeloid Cell Integrin α4β1, Leading to Tumor Inflammation and Growth

**DOI:** 10.1371/journal.pone.0060226

**Published:** 2013-04-02

**Authors:** Michael C. Schmid, Irene Franco, Sang Won Kang, Emilio Hirsch, Lawrence A. Quilliam, Judith A. Varner

**Affiliations:** 1 Moores Cancer Center, University of California San Diego, La Jolla, California, United States of America; 2 Departments of Genetics, Biology and Biochemistry, Molecular Biotechnology Center, School of Medicine, University of Torino, Italy; 3 Division of Life and Pharmaceutical Science, Ewha Womans University, Seoul, Korea; 4 Department of Biochemistry and Molecular Biology, Indiana University of School of Medicine, Indianapolis, Indiana, United States of America; 5 Departments of Pathology and Medicine, University of California San Diego, La Jolla, California, United States of America; University of Bergen, Norway

## Abstract

Tumor inflammation, the recruitment of myeloid lineage cells into the tumor microenvironment, promotes angiogenesis, immunosuppression and metastasis. CD11b+Gr1lo monocytic lineage cells and CD11b+Gr1hi granulocytic lineage cells are recruited from the circulation by tumor-derived chemoattractants, which stimulate PI3-kinase γ (PI3Kγ)-mediated integrin α4 activation and extravasation. We show here that PI3Kγ activates PLCγ, leading to RasGrp/CalDAG-GEF-I&II mediated, Rap1a-dependent activation of integrin α4β1, extravasation of monocytes and granulocytes, and inflammation-associated tumor progression. Genetic depletion of PLCγ, CalDAG-GEFI or II, Rap1a, or the Rap1 effector RIAM was sufficient to prevent integrin α4 activation by chemoattractants or activated PI3Kγ (p110γCAAX), while activated Rap (RapV12) promoted constitutive integrin activation and cell adhesion that could only be blocked by inhibition of RIAM or integrin α4β1. Similar to blockade of PI3Kγ or integrin α4β1, blockade of Rap1a suppressed both the recruitment of monocytes and granulocytes to tumors and tumor progression. These results demonstrate critical roles for a PI3Kγ-Rap1a-dependent pathway in integrin activation during tumor inflammation and suggest novel avenues for cancer therapy.

## Introduction

The link between cancer and inflammation is increasingly recognized; at least fifteen percent of cancer cases arise in association with chronic inflammation, such as those caused by infectious agents (helicobacter pylori/gastric cancer, hepatitis C/liver cancer, and papilloma virus/cervical cancer), environmental toxins (asbestos, coal dust, and tobacco smoke), autoimmune disorders (Crohn’s disease) and potentially obesity (1–3). Extensive infiltration of tissues by immunosuppressive macrophages is a common element of inflammatory diseases and tumors (4–6). In chronically inflamed tissues and tumors, among the most populous inflammatory cells are macrophages (TAMs), which express numerous factors that can stimulate angiogenesis, metastasis, inflammation and immunosuppression, as well as relapse after therapy (4–16). Targeting the causes and consequences of chronic inflammation is likely to provide significant benefit in the treatment and prevention of a wide variety of cancers. Thus, identification of the common mechanisms controlling inflammatory cell recruitment to tumors is a promising approach to suppress tumor growth and progression.

**Figure 1:**
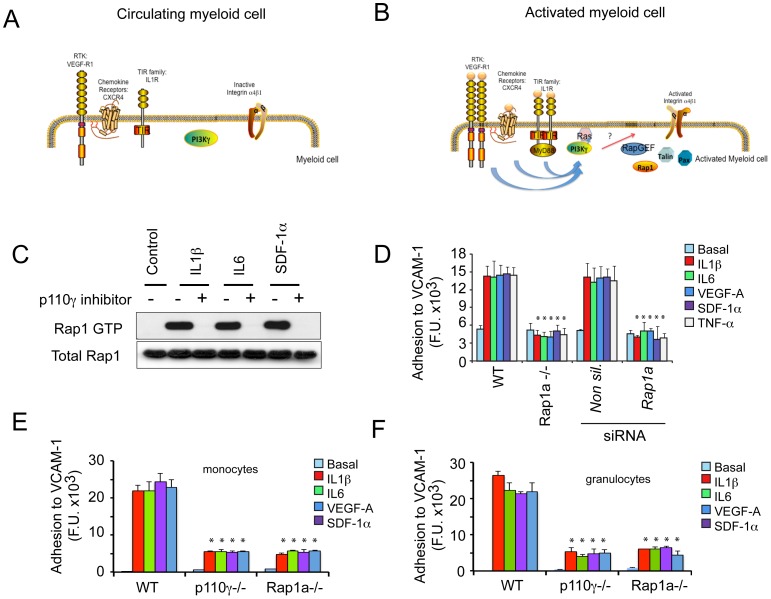
Rap1a and PI3Kγ each promote integrin α4β1-dependent myeloid cell adhesion. (A) Schematic of circulating, unstimulated myeloid cells, indicating inactive receptor tyrosine kinases, chemokine receptors and Toll-like Receptor/IL-1 Receptor family members (TIR family) and inactive downstream PI3Kγ and integrin α4β1. (B) Schematic of stimulated myeloid cells showing all three receptor types that activate PI3Kγ, thereby promoting integrin α4β1 conformational changes, leading to integrin activation. Co-factors that may play roles in activating integrin α4β1 include PKC, Rap1, RIAM, talin and paxillin. (C) Representative immunoblot of GTP-Rap and total Rap in myeloid cells that were stimulated with or without the chemoattractants IL-1β, IL6 or SDF1α and treated with the PI3Kγ inhibitor, TG100-115 (+) or the chemically similar, inert control (-). (D) Adhesion to the integrin α4β1 ligand VCAM-1 (expressed as fluorescence units, F.U.) of chemoattractant-stimulated WT and Rap1a−/− CD11b+ cells and Rap1a or non-silencing siRNA-transfected CD11b+ cells (n = 3), *P<0.01, Rap1a−/− vs WT and Rap1a vs nonsilencing siRNA. (E) Adhesion to VCAM-1 of CD11bGr1lo monocytes sorted from WT, p110γ−/− and Rap1a−/− animals (n = 3), *P<0.01, p110γ−/− and Rap1a−/− vs WT. (F) Adhesion to VCAM-1 of CD11bGr1hi granulocytes sorted from WT, p110γ−/− and Rap1a−/− animals (n = 3), *P<0.01, p110γ−/− and Rap1a−/− vs WT.

Diverse chemoattractants recruit innate immune cells to chronically inflamed tissues and tumors; these can activate G protein coupled receptors (GPCR), receptor tyrosine kinases (RTK) or Toll-like/interleukin-1 receptors (TLR/IL1R) to initiate myeloid cell recruitment (9–10, 12–15). We recently showed that each of these receptors stimulate myeloid cell recruitment by activating PI3-kinase γ, but not α, β or δ, in circulating myeloid cells (9). It is well-accepted that Class IA PI3K isoforms p110α, β and δ can be activated downstream of RTKs through the engagement of the regulatory p85 subunit by receptor phosphotyrosines. In contrast, the Class IB isoform p110γ can be activated by GPCRs via the β-γ subunits of heterotrimeric G proteins. We recently demonstrated that whereas GPCRs activate p110γ in a Ras/p101-dependent manner, RTKs and TLR/IL1Rs directly activated p110γ in a Ras/p87-dependent manner. Once activated, p110γ promoted inside-out activation of integrin, α4β1, causing granulocytic and monocytic cell adhesion to endothelium and invasion into tumors (Figure 1A–B). Pharmacological or genetic blockade of p110γ suppressed recruitment of both monocytes and granulocytes, and suppressed angiogenesis, tumor growth, progression and metastasis of implanted and spontaneous tumors, revealing an important therapeutic target in oncology (10). These findings indicated that targeting the molecular mechanisms promoting the recruitment of myeloid cells during inflammation could provide significant benefit in the treatment of a wide variety of cancers.

The integrin family of adhesion proteins plays a significant role in regulating inflammation (17–19). Activation of integrin α4β1 by inside-out signaling induced by chemoattractants is required for lymphocyte and myeloid cell extravasation from the vasculature (9–10, 18–20). Extracellular stimuli from chemoattractants promote conformational changes in integrin extracellular domains that increase their capacity to bind ligand and therefore attach to endothelium and extracellular matrices (21–22). Although all the steps in integrin activation have yet to be deciphered, integrin activation can depend on Rap1 (Ras-proximate-1), a Ras-like small GTP-binding protein (23–25). Rap1 activates the effector protein, RIAM, which interact closely with the cytoskeletal protein talin, thereby localizing it near the membrane (26–27). Talin binds to sites in integrin β chain cytoplasmic tails, thereby disrupting electrostatic interactions between amino acids in the integrin α and β chain cytoplasmic tails and altering the packing of integrin transmembrane domains (28–30). These events alter the conformation of the extracellular domains of the integrin heterodimer and increase ligand binding. In addition, paxillin-binding to the integrin α4 cytoplasmic tail also promotes integrin activation, as disruption of the paxillin-binding site in the integrin α4 cytoplasmic tail partially prevents talin binding and inhibits adhesion and trafficking of lymphocytes and myeloid cells (20, 31). Our recent studies showed that PI3Kγ activation by a variety of inflammatory stimuli is essential to promote integrin α4 conformational changes, cell adhesion and myeloid cell trafficking in vivo (9–10). However, the exact molecular mechanisms by which myeloid cell PI3Kγ regulates integrin α4β1 activation in vitro and in vivo remained to be elucidated. Here we show that PI3Kγ-mediated integrin activation and subsequent myeloid cell recruitment and tumor inflammation depends on PLCγ, CalDAG-GEFI and II, Rap1a and RIAM.

## Results

### PI3Kγ Activates Integrin α4β1 in a Rap1a-dependent Manner

As we recently showed that integrin α4β1 activation in CD11b+ granulocytes and monocytes depends on PI3Kγ (p110γ) (9–10), we sought to determine how PI3Kγ activates integrin α4β1. Integrin activation has been shown to depend on members of the Rap1 family of small GTPases (23–25). Therefore, we asked whether myeloid cell PI3Kγ could promote integrin α4β1 activation in a Rap1a-dependent manner. Like other small GTPases, Rap1 cycles between GDP-bound inactive and GTP-bound active forms. Thus, we measured Rap1 activity in CD11b+ bone marrow derived myeloid cells using a Ral-RBD (Ras Binding Domain) pull-down assay, which measures GTP-bound Rap in cell lysates. We found that the chemoattractants IL-1β, IL-6, and SDF-1α stimulated Rap-GTP loading in CD11b+ myeloid cells, but Rap-GTP loading was inhibited in the presence of the p110γ inhibitor TG100-115 (Figure 1C). To determine whether integrin α4β1 activation in myeloid cells depends on Rap1a, we evaluated the capacity of Rap1a deficient (Rap1a−/−) and Rap1a siRNA transfected WT myeloid cells to adhere to VCAM-1. While Rap1a-deficient mice develop with minimal defects, leukocytes from these mice have exhibited reduced responsiveness to chemoattractants (33–36). Indeed, Rap1a−/− and Rap1a siRNA transfected CD11b+ bone marrow derived myeloid cells exhibited significantly reduced adhesion to VCAM-1 in response to a variety of chemoattractants (Figure 1D), even though Rap1a deficient myeloid cells expressed normal levels of integrin α4β1 (Figure S1A–B). The geranylgeranyltransferase inhibitor GGTI-2147, which prevents the post-translational modification required to target small GTPases such as Rap1 to membranes, also effectively blocked chemoattractant-induced myeloid cell adhesion (Figure S1C). Importantly, p110γ−/− and Rap1a−/− bone marrow derived CD11bGr1lo monocytes (Figure 1E) and CD11bGr1hi granulocytes (Figure 1F) failed to adhere significantly to VCAM-1. Taken together, these data suggest that p110γ-mediated activation of integrin α4β1 in myeloid cells depends on Rap1a.

**Figure 2:**
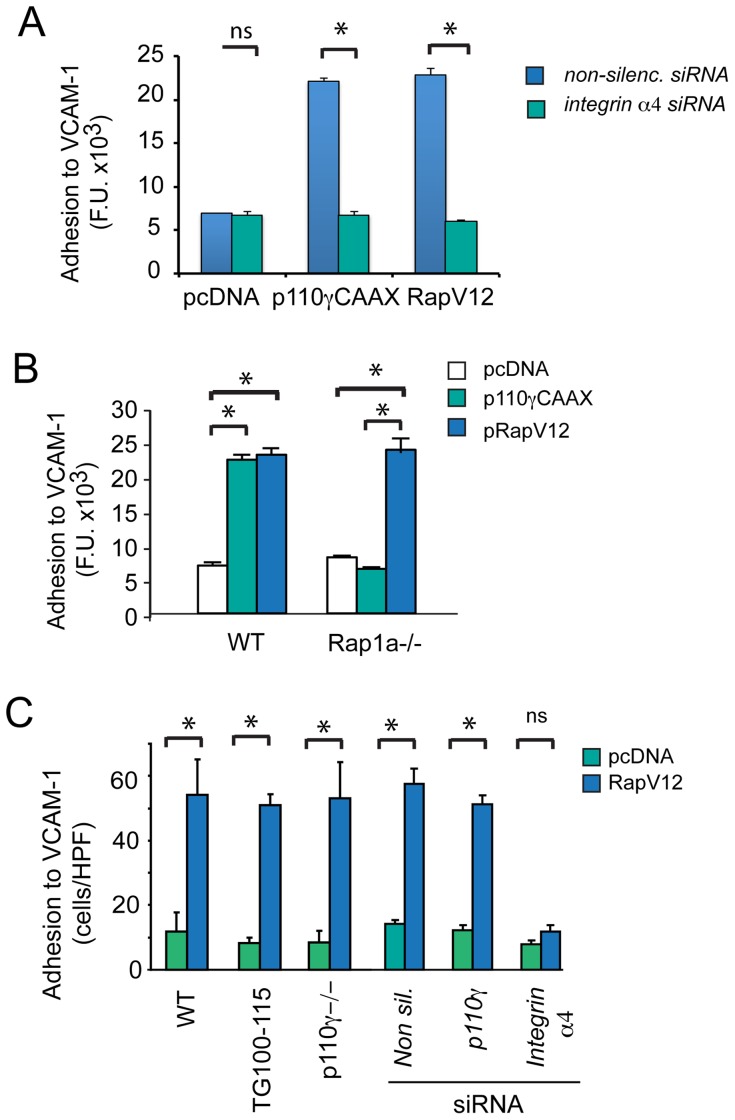
PI3Kγ-mediated integrin α4β1 adhesion requires Rap1a. (A) Adhesion to VCAM-1 of p110γCAAX, pRapV12 or pcDNA expressing CD11b+ myeloid cells that were also transfected with either integrin α4 or control siRNA, *P<0.01, integrin α4 vs non-silencing siRNA (n = 3). (B) Adhesion to VCAM-1 of p110γCAAX, pRapV12 and pcDNA (control transfected) WT or Rap1a−/− CD11b+ myeloid cells (n = 3), *P<0.01, p110γCAAX or pRapV12 vs pcDNA. (c) Adhesion to VCAM-1 of pRapV12 and pcDNA chemoattractant-stimulated WT, TG100-115 (PI3Kγ inhibitor)-treated, p110γ−/−, non-silencing, p110γ and integrin α4 siRNA-transfected CD11b+ myeloid cells (n = 3), *P<0.01 Rap1a vs pcDNA.

To determine whether Rap1a is sufficient for p110γ-mediated integrin α4β1 activation in myeloid cells, we expressed constitutively activated PI3Kγ (p110γCAAX), constitutively activated Rap1 (RapV12) or control plasmid (pcDNA) in CD11b+ myeloid cells. Constitutively activated p110γ and Rap1 both stimulated adhesion to VCAM-1 in myeloid cells even in the absence of added chemoattractants. However, integrin α4-targeted siRNA blocked p110γCAAX and RapV12 stimulated myeloid cell adhesion to VCAM-1, indicating that p110γ and Rap1 are both upstream of integrin α4β1 (Figure 2A, Figure S1D–E). Importantly, expression of constitutively activated Rap1, but not activated p110γ, promoted adhesion in Rap1a−/− myeloid cells (Figure 2B). Furthermore, RapV12 induced constitutive myeloid cell adhesion in both WT and p110γ−/− myeloid cells and in TG100-115 and p110γ siRNA treated cells (Figure 2C), indicating that p110γ is upstream of Rap1a in the integrin activation pathway. Taken together, our results indicate that Rap1a is activated by PI3Kγ and is both necessary and sufficient to promote PI3Kγ-mediated integrin α4-dependent myeloid cell adhesion.

### Rap1a Promotes Integrin α4β1 Conformational Changes and Ligand Binding

**Figure 3:**
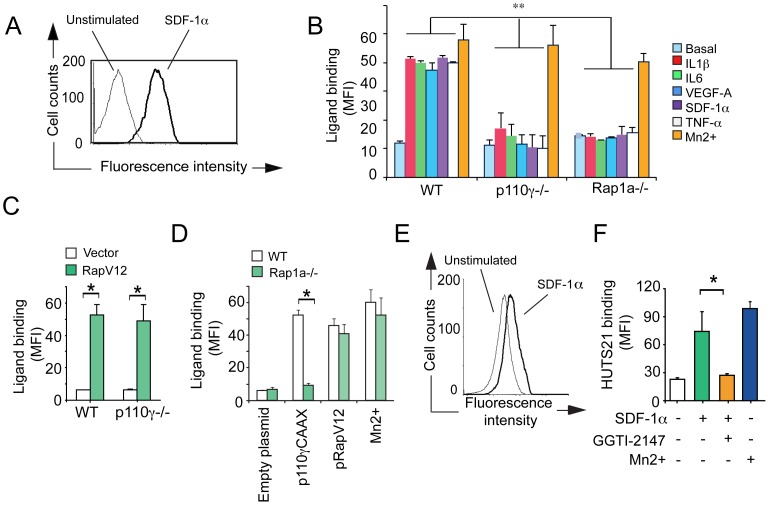
Rap1 promotes myeloid cell integrin α4β1-ligand binding and conformational changes. (A) Representative histogram of soluble, fluorescently labeled VCAM-1/Fc bound to CD11b+ myeloid cells 3 min after treatment with (black line) or without (grey line) IL-1β. (B) Mean fluorescence intensity (MFI) of VCAM-1/Fc bound to WT, p110γ−/− and Rap1a−/− myeloid cells in the absence (basal) or presence of chemoattractants or the positive control stimulus Mn2+ (n = 3), *P<0.01, p110γ−/− or Rap1a−/− vs WT. (C) MFI of VCAM-1/Fc bound to pRapV12- and control-transfected WT and p110γ−/− myeloid cells (n = 3), *P<0.01, pRapV12 vs control. (D) MFI of VCAM-1/Fc bound to WT and Rap1a−/− myeloid cells transfected with p110γCAAX, pRapV12 or control plasmid or treated with Mn2+ (n = 3), *P<0.01, Rap1a−/− vs WT. (E) Representative histogram of HUTS21 antibody binding to unstimulated (grey line) or SDF-1α stimulated (black line) human CD11b+ myeloid cells. (F) MFI of HUTS21 binding to unstimulated or SDF-1α stimulated human myeloid cells in the presence of absence of 10 µM geranylgeranyltransferase inhibitor (GGTI-2147) or Mn2+ (n = 3), *P<0.01, +GGTI vs -GGTI.

Extracellular stimuli induce integrin conformational changes that result in increased ligand-binding and cell adhesion, a process that is called “integrin activation”. Chemoattractants rapidly (within seconds) induce PI3Kγ-dependent myeloid cell integrin β1 conformational changes, as measured by cell surface binding of HUTS21, an antibody that recognizes an epitope expressed only on activated human β1 integrin (10, 22). These chemoattractants also rapidly stimulate the binding of recombinant VCAM-1 to myeloid cell integrin α4β1 in WT but not PI3Kγ or integrin α4 deficient myeloid cells (10). To determine whether Rap1a is required for PI3Kγ-mediated integrin α4β1 conformational changes, chemoattractant-stimulated myeloid cells from WT, p110γ−/− and Rap1a−/− mice were incubated with soluble VCAM-1/Fc. Bound VCAM-1/Fc was then detected with fluorescently-labeled antibodies (Figure 3A). Chemoattractants markedly stimulated the binding of soluble VCAM-1/Fc to WT but not PI3Kγ−/− or Rap1a−/− myeloid cells (Figure 3B). Mn2+, which binds to the integrin-ligand binding pocket and directly promotes ligand binding and thus integrin activation, stimulated VCAM-1/Fc binding to WT, PI3Kγ−/− and Rap1a−/− cells, demonstrating that integrin α4β1 is capable of being activated in all three genotypes. In support of these findings, we found that pharmacological blockade of Rap1 by GGTI-2147 suppressed chemoattractant-induced VCAM-1-binding to myeloid cells (Figure S2). Importantly, expression of activated RapV12 restored ligand binding to p110γ−/− (Figure 3C) and Rap1a−/− myeloid cells (Figure 3D). As expression of activated PI3Kγ (p110γCAAX) failed to stimulate ligand binding in Rap1a−/− myeloid cells (Figure 3D), these results further support the concept that Rap1a is downstream of PI3Kγ and is essential for PI3Kγ-stimulated ligand binding to integrin α4β1.

We also found that while chemoattractants stimulated binding of HUTS21 on human myeloid cells, the Rap1 selective inhibitor GGTI-2147 inhibited HUTS21 binding, confirming that integrin activation and conformational changes depend on Rap1 (Figure 3E–F). Taken together, our results show that Rap1a is necessary and sufficient to activate integrin α4β1 in both mouse and human myeloid cells.

### CalDAG-GEFs and PLCγ are Required for PI3Kγ Mediated Integrin Activation

**Figure 4:**
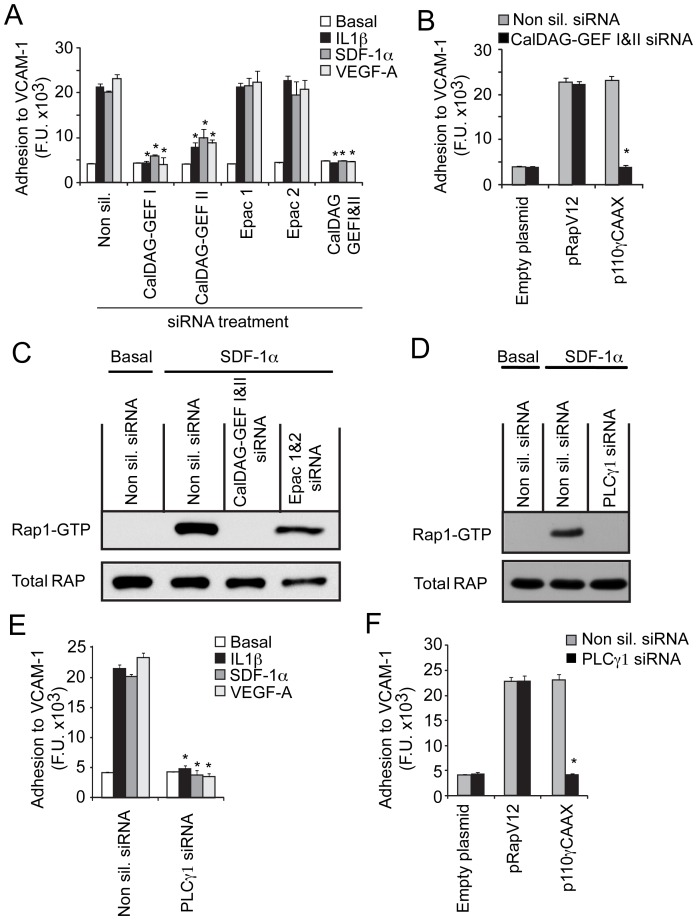
CalDAG-GEFs are required for PI3Kγ-mediated Rap1 and integrin α4β1 activation in myeloid cells. (A) Adhesion of chemoattractant-treated CD11b+ myeloid cells to VCAM-1 after transfection with non-silencing, CalDAG-GEFI, CalDAG-GEFII, Epac1, Epac2 and CalDAG-GEFI+CalDAG-GEFII siRNAs (n = 3), *P<0.01, CalDAG-GEFI, CalDAG-GEFII vs non silencing siRNA. (B) Adhesion to VCAM-1 of myeloid cells after transfection with CalDAG-GEFI and II or control siRNAs in combination with pRapV12, p110γCAAX, or empty plasmid (n = 3), *P<0.01 CalDAG-GEFI and II vs control siRNAs. (C) Rap1 GTP and total Rap immunoblot of unstimulated, non-silencing siRNA-transfected (Basal) myeloid cells and SDF-1α stimulated CalDAG-GEFI and II, Epac 1 and 2, and non-silencing siRNA-transfected myeloid cells. (D) Rap1 GTP and total Rap immunoblot of unstimulated non-silencing siRNA-transfected (basal) and SDF-1α-stimulated myeloid cells after transfection with PLCγ1 or control siRNA. (E) Adhesion of PLCγ1 or control siRNA-transfected, chemoattractant-stimulated myeloid cells (n = 3), *P<0.01 PLCγ1 vs non-silencing siRNA. (F) Adhesion to VCAM-1 of myeloid cells after transfection with PLCγ1 or control siRNA in combination with pRapV12, p110γCAAX, or vector control (empty plasmid) (n = 3), *P<0.01 vs PLCγ1 non-silencing siRNA.

As it is not clear how PI3Kγ activates Rap1a, we postulated that PI3Kγ might activate Rap1a exchange factors. Guanine nucleotide exchange factors (GEFs) exchange GDP for GTP to generate the active form of monomeric GTPase (37). As some RapGEFs are ubiquitously expressed while others are expressed only in select tissues (25), we examined the expression of RapGEFs in primary murine myeloid cells and found that four RapGEFs, CalDAG-GEFI/RasGRP2, CalDAG-GEFII/RasGRP, Epac1 and Epac2 (37–40) are expressed in primary bone marrow derived myeloid cells. While all four GEFs were expressed, mRNA levels of Epac1 were most abundant (Supplementary Figure 3A). To determine if any of these RapGEFs play a role in integrin activation, we suppressed their gene expression in myeloid cells by siRNA mediated gene knockdown. siRNA knockdown of CalDAG-GEFI or II but not Epac1 or 2 blocked chemoattractant-induced integrin α4β1-mediated cell adhesion (Figure 4A; Figure S3B). Combined inhibition of both CalDAG-GEFI and II blocked myeloid cell adhesion to the same extent as blockade of CalDAG-GEFI alone, suggesting that CalDAG-GEFI may play a primary role in PI3Kγ-mediated Rap1a activation (Figure 4A). Inhibition of CalDAG-GEFI and II together blocked myeloid cell adhesion induced by p110γCAAX but did not inhibit adhesion induced by pRapV12, indicating that RapGEFs act upstream of Rap1 in integrin activation (Figure 4B). As CalDAG-GEFI knockout mice also exhibit defects in leukocyte integrin activation (40–41), these results indicate that CalDAG-GEFI and II promote PI3Kγ-mediated adhesion but are not required once Rap has been activated. Importantly, knockdown of CalDAG-GEFI and CalDAG-GEFII, but not Epac1 and 2, prevented Rap1 GTP-loading in response to SDF1α (Figure 4C). These findings show that the myeloid cell GEFs CalDAG-GEFI and CalDAG-GEFII activate Rap1 and thereby promote integrin α4β1-mediated adhesion in response to PI3Kγ activation.

CalDAG-GEFs can be activated by calcium or diacylglycerol (DAG), two second messengers produced by the actions of phospholipase Cγ (PLCγ) (42). Each PLC isoform contains a plextrin-homology domain, which allows it to bind to phosphatidyl inositol in the plasma membrane (43). Importantly, as PI(P3,4,5)P_3_ can activate PLCγ (44), it is possible that PI3Kγ activates Rap1 by stimulating PLCγ in myeloid cells. Therefore, we asked whether PLCγ is necessary for Rap1 activation in myeloid cells. Two isoforms of PLCγ are expressed in primary myeloid cells, PLCγ1 and PLCγ2. We found that siRNA-mediated knockdown of PLCγ1 blocked Rap1 GTP-loading induced by SDF-1α stimulation (Figure 4D). siRNA-mediated knockdown of PLCγ1 (Figure 4E–F, Figure S3C) or pharmacological inhibition of PLCγ (Figure S3D) completely inhibited myeloid cell adhesion to VCAM-1 in response to stimulation by chemoattractant factors or expression of activated PI3Kγ (p110γCAAX) (Figure 4E–F). As knockdown of PLCγ1 expression does not block adhesion in pRapV12 expressing cells (Figure 4F), our results indicate that PLCγ1 functions downstream of PI3Kγ and upstream of Rap1a. PLCγ1 thus is required for PI3Kγ activation of Rap1a and α4β1 dependent myeloid cell adhesion. Although calcium and diacylglycerol can activate some isoforms of protein kinase C (45), we observed no role for PKC in integrin activation in myeloid cells, as neither broad spectrum nor selective PKC inhibitors affected myeloid cell adhesion to VCAM-1 (Figure S4A–B).

**Figure 5:**
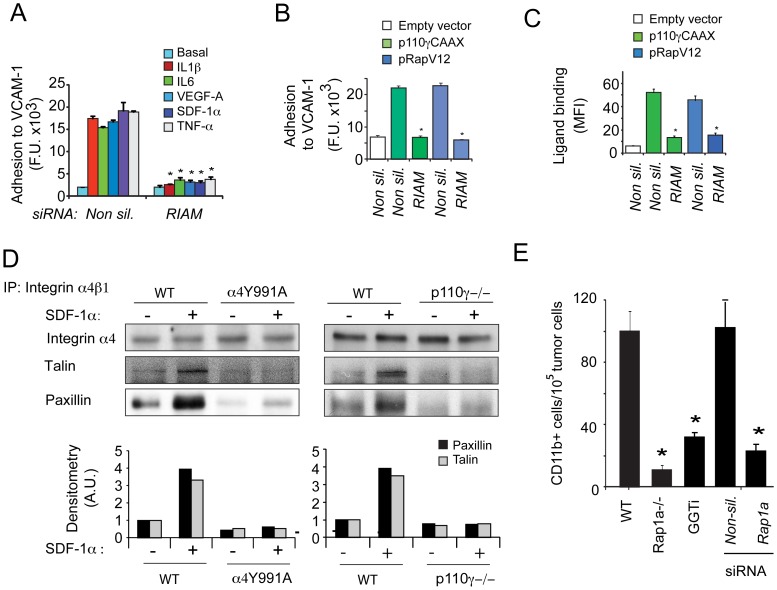
RIAM is necessary for PI3Kγ-mediated integrin α4β1 activation. (A) Adhesion to VCAM-1 of chemoattractant-treated myeloid cells after transfection with RIAM or control siRNA (n = 3), *P<0.01 vs RIAM vs. non-silencing siRNA. (B) Adhesion to VCAM-1 of RIAM or non-silencing siRNA-transfected myeloid cells that were also transfected with p110γCAAX, pRapV12 or control plasmid (n = 3), *P<0.01 RIAM vs non-silencing siRNA (C) VCAM-1/Fc binding to RIAM or non-silencing siRNA-transfected myeloid cells that were also transfected with p110γCAAX, pRapV12 or control plasmid (n = 3), *P<0.01 RIAM vs non-silencing siRNA. (D) Immunoprecipitates of integrin α4β1 from WT, α4Y991A, and p110γ−/− bone marrow derived cells with (+) or without (−) SDF-1α stimulation and immunoblotted to detect the integrin α4 subunit, talin, and paxillin. Histograms: densitometry of talin and paxillin levels normalized to integrin α4 levels. (E) Trafficking to LLC tumors of WT or Rap1a−/− myeloid cells, myeloid cells treated with 10 µM GGTI-2147, and myeloid cells transfected with Rap1a or non-silencing siRNAs, (n = 3), *P<0.01 Rap1a−/− or 10 µM GGTI-2147 vs WT and Rap1a vs non-silencing siRNAs.

Integrin α4-mediated myeloid cell adhesion depends on the interaction of the adapter proteins talin and paxillin with the cytoplasmic tails of integrin α4β1 (9,20,31). Previous studies implicated the Rap1 effector RIAM in facilitating talin binding to the integrin β cytoplasmic tail and immune cell adhesion events (26–28). As the Rap1 effector RIAM is strongly expressed in myeloid cells, to determine whether RIAM promotes integrin α4β1 activation, we evaluated the effect of siRNA-mediated knockdown of RIAM on myeloid cell adhesion. RIAM siRNA blocked cell adhesion in response to chemoattractants (Figure 5A, Figure S4C) and expression of activated Rap1 or PI3Kγ (Figure 5B). Moreover, inhibition of RIAM expression prevented integrin α4β1 ligand-binding in response to p110γCAAX or RapV12 expression (Figure 5C), suggesting that RIAM directly regulates integrin α4 activation and myeloid cell adhesion downstream of both PI3Kγ and Rap1. Our studies thus indicate that RIAM is required for integrin α4 activation in myeloid cells.

As RIAM promotes integrin activation by facilitating interaction of talin with the integrin β1 cytoplasmic tail (26), we next asked whether PI3Kγ activation promotes talin and paxillin binding to integrin α4β1. To accomplish this goal, we immunoprecipitated integrin α4β1 from unstimulated and chemoattractant stimulated WT, α4Y991A, and PI3Kγ−/− cells and determined whether talin and paxillin co-immunoprecipitated with the integrin. We found that talin and paxillin co-immunoprecipitated with integrin α4β1 in stimulated WT but not in stimulated α4Y991A or p110γ−/− myeloid cells (Figure 5D). Taken together, these findings indicate that Rap1 and its effector RIAM facilitate integrin α4β1 activation in response to PI3Kγ activation by stimulating association of the adapter proteins talin and paxillin with integrin α4β1.

In previous studies, we found that PI3Kγ-mediated integrin α4β1 activation is required for trafficking of myeloid cells to the tumor microenvironment (10). To investigate whether Rap1a is required for this recruitment of myeloid cells to the tumor microenvironment, we adoptively transferred fluorescently labeled myeloid cells into mice bearing established subcutaneous Lewis lung carcinoma (LLC) tumors and within 2 h, we could quantitatively identify these cells in tumors using flow cytometry. We found that Rap1a−/−, Rap1a siRNA transfected and GGTI-2147 treated myeloid cells failed to accumulate in the tumor microenvironment (Figure 5E), indicating that Rap1a, like p110γ and integrin α4β1 (9–10), is required for myeloid cell trafficking to tumors.

**Figure 6:**
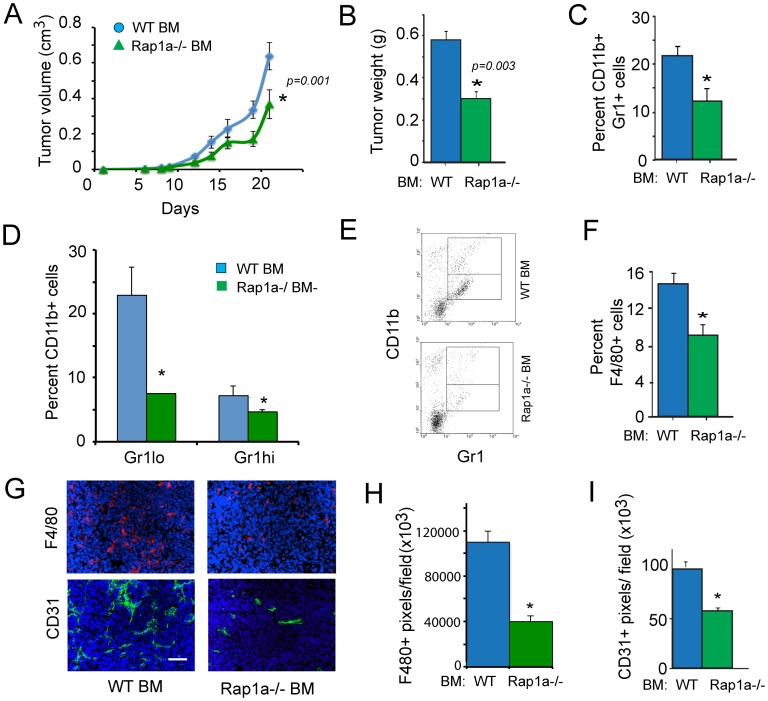
Myeloid cell Rap1a promotes tumor inflammation and growth. (A) Tumor volume and (B) weight of LLC tumors grown in WT mice transplanted with WT or Rap1a−/− bone marrow (n = 10); *P = 0.001 to 0.003, WT vs Rap1a−/− BM, as determined by ANOVA. (C) Percentage of CD11b+Gr1+ tumor-infiltrating myeloid cells in WT and Rap1a−/− bone marrow transplanted (BMT) tumors, *P<0.01, Rap1a−/− vs WT (ANOVA). (D) Percentage of CD11b+Gr1lo monocytic and CD11b+Gr1hi granulocytic lineage cells in WT and Rap1a−/− BMT tumors, *P<0.01 Rap1a−/− vs WT (ANOVA). (E) Representative FACs profiles from D, with upper quadrant corresponding to CD11b+Gr1hi granulocytic and lower quadrant corresponding to CD11b+Gr1lo monocytic lineage cells. (F) Percentage of F4/80+ tumor-infiltrating myeloid cells in WT and Rap1a−/− BMT tumors, *P<0.01 Rap1a−/− vs WT (ANOVA). (G) Representative images of tumor cryosections that were immunostained to detect F4/80+ macrophages (red) and CD31+ blood vessels (green) and counterstained with Dapi. Scale bar = 40 µM. (H) Quantification of F4/80+ pixels/field in cryosections from G, *P<0.01 Rap1a−/− vs WT. (I) CD31+ pixels/field in cryosections from G, *P<0.01 Rap1a−/− vs WT.

To determine the impact of myeloid cell Rap1a on tumor inflammation and progression, we evaluated the growth of LLC tumors in WT mice that were lethally irradiated and transplanted with bone marrow from WT or Rap1a−/− mice. Importantly, mice transplanted with Rap1a−/− BM exhibited significantly reduced tumor growth (Figure 6A–B). Tumors from mice with Rap1a−/− bone marrow showed significant reductions in the recruitment of total CD11b+Gr1+ myeloid cells (Figure 6C), in the recruitment of monocytic and granulocytic subpopulations (Figure 6D–E) and CD11b+F4/80+ macrophages (Figure 6F) to the tumor microenvironment, as determined by protease-mediated dissolution of tumors and flow cytometric quantification of cell subpopulations. Similar reductions in tumor growth and inflammation were also observed in Rap1a−/− animals (Figure S5A–D). We also observed reduced recruitment of macrophages and blood vessels in mice with Rap1a−/− bone marrow cells (Figure 6G–I). Together, these results indicate that Rap1a is an important mediator of tumor inflammation and progression through its actions on integrin activation during the recruitment of myeloid cells to the tumor microenvironment, with subsequent effects on tumor angiogenesis.

## Discussion

**Figure 7:**
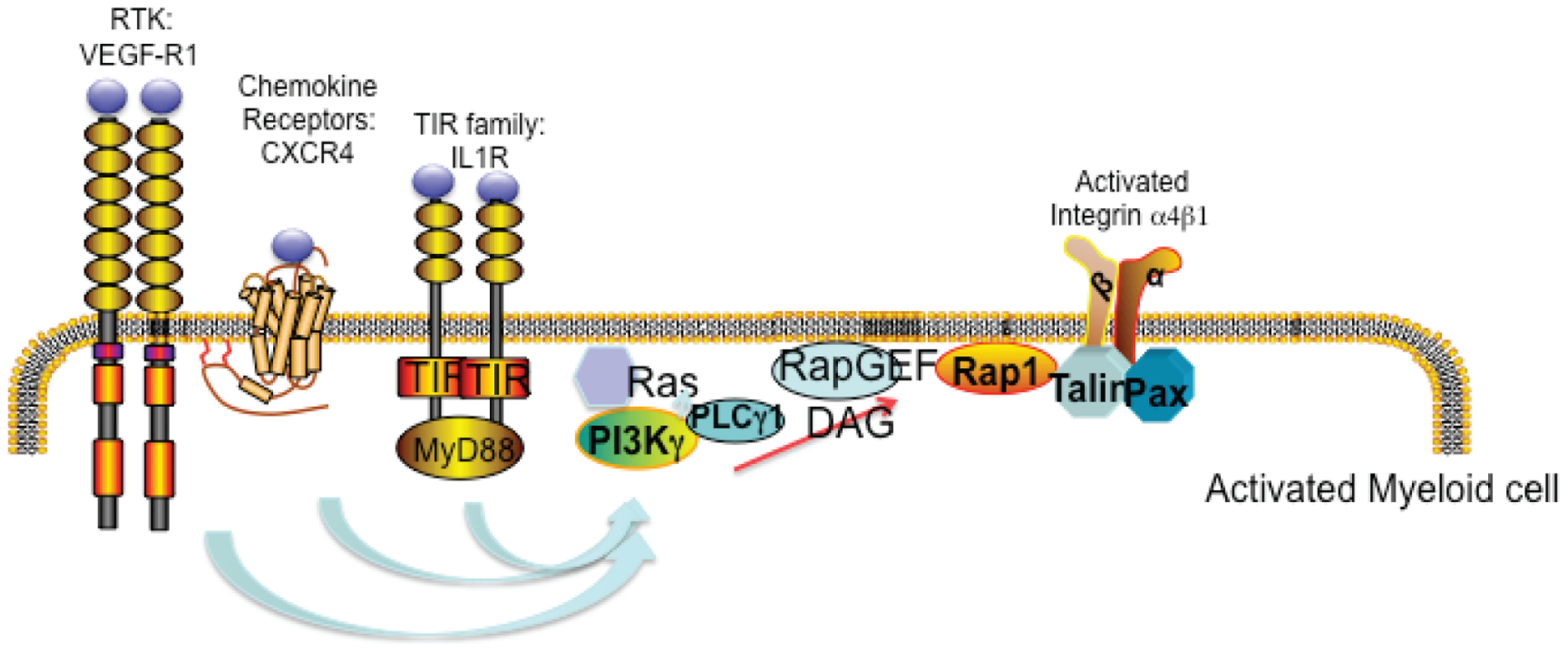
PI3Kγ mediated activation of integrin α4β1 requires Rap1a and other co-factors. Schematic of stimulated myeloid cells demonstrating that diverse receptor types activate PI3Kγ, thereby promoting integrin α4β1 conformational changes, leading to integrin activation in a PLCγ1, RAP-GEF, Rap1, RIAM, talin and paxillin-dependent manner.

Tumor inflammation promotes angiogenesis, immunosuppression and metastasis. Targeting the causes and consequences of chronic inflammation is likely to provide significant benefit in the treatment and prevention of a wide variety of cancers. Our previous studies have investigated the mechanisms demonstrated that myeloid derived suppressor cells are recruited from the circulation by tumor-derived chemoattractants, which stimulate PI3-kinase γ (PI3Kγ)-mediated integrin α4 activation and myeloid cell extravasation into tumors (9). We have show here that PI3Kγ activates PLCγ, leading to RasGrp/CalDAG-GEF-I&II mediated, Rap1a-dependent activation of integrin α4β1, extravasation of myeloid cell, and inflammation-associated tumor progression. Our studies demonstrate that chemoattractants stimulate myeloid cell RTK, GPCR and TIR-receptor-mediated integrin α4β1 activation, myeloid cell tumor trafficking and tumor inflammation and growth by promoting PI3Kγ-dependent Rap1a activation. PI3Kγ mediated Rap1a and integrin α4β1 activation depend on PLCγ, Rap-GEFs CalDAG-GEFI and II and RIAM. RIAM promotes integrin α4β1 conformational changes through its interactions with talin. Talin then disrupts the packing of the integrin cytoplasmic tails, leading to integrin unfolding and activation (Figure 7). In vivo, tumor-derived chemoattractants promote the recruitment and infiltration of myeloid cells to the tumor microenvironment by increasing PI3Kγ-Rap1a-integrin α4β1-dependent adhesion to the tumor endothelium. As myeloid cells in the tumor microenvironment suppress T-cell mediated anti-tumor immunity (3–5), promote tumor angiogenesis (7–13), and contribute to resistance to chemotherapy (9), our studies thus suggest novel targets for the development of therapeutic approaches for cancer and inflammatory diseases.

Tumors release a diverse array of chemoattractants that promote myeloid cell infiltration of tumors; these chemoattractants activate G protein coupled receptors, receptor tyrosine kinases or Toll-like/interleukin-1 receptors to initiate myeloid cell recruitment (9–10,12–15). Using competitive inhibitors of chemoattractants and PI3K genetic mutations, siRNA-mediated knockdown and chemical inhibitors of PI3K isoforms, we recently showed that a variety of tumor-derived chemoattractants stimulate myeloid cell recruitment by activating PI3Kγ, but not other PI3K isoforms, in circulating myeloid cells (9–10). We showed here that PI3Kγ activity alone is sufficient to promote integrin α4β1 activation, as expression of membrane targeted PI3Kγ (p110γCAAX) alone increases myeloid cell adhesion and α4 ligand binding. Our studies have shown that in primary myeloid cells and macrophages, PI3Kγ is the only PI3K isoform that is expressed at significant levels. However, preliminary studies indicate that myeloid leukemia cells exhibit higher levels of other PI3K isoforms, such as PI3Kδ, than PI3Kγ (not shown). These cells exhibit constitutive adhesion to VCAM-1, suggesting that once activated, other PI3K isoforms can promote integrin α4β1 activation. Using p110γCAAX expression, we were able to show that once activated, PI3Kγ is sufficient to activate integrin α4β1 and that this event depends on Rap1 activity.

Two Rap isoforms, Rap1a and Rap1b, have been implicated in the regulation of integrin activation (33–36, 47). Rap1b has a clear role in regulating platelet-mediated integrin αIIbβ3 activation (36), while Rap1a has been implicated in regulating leukocyte and endothelial cell migration (33–35)^.^ In the studies described here, we found that Rap1a is required to promote integrin α4β1 activation and adhesion. Our prior studies showed that co-localization of integrin α4β1, N/K Ras, p110γ and specific PI3K regulatory subunits in plasma membranes is required for integrin α4β1 activation in response to inflammatory signals. Our current results suggest that the Rap1a isoform may also co-localize in these membrane microdomains to promote integrin α4β1 activation in myeloid cells.

In the studies described here, we found that Rap1a is required to promote integrin α4β1-dependent myeloid cell trafficking during inflammation and tumor growth in vivo, as we observed substantial suppression of tumor growth and inflammation in mice transplanted with Rap1a−/− bone marrow. Although several reports have shown distinct functional roles within cells for Rap1a and Rap1b (34, 36, 48), knockdown of Rap1a in primary myeloid cells resulted in almost complete loss of PI3Kγ-mediated integrin α4β1 activity in vitro, suggesting that Rap1a is the predominant form of Rap1 that is activated by PI3Kγ in these cells.

Our studies demonstrate that two RapGEFs, CalDAG-GEFI and CalDAG-GEFII, but not Epac 1 or 2, promote integrin α4β1 activation in myeloid cells. Importantly, mice and patients lacking CalDAG-GEFI exhibit leukocyte adhesion deficiencies (40–41) as do mice lacking Rap1a (33). Although Epac1, a cAMP-activated GTP exchange factor (38–39), is the most highly expressed RapGEF in myeloid cells, we found that it plays no role in PI3Kγ-meditated integrin activation, as siRNA-mediated knockdown of Epac1 did not affect integrin activation or myeloid cell adhesion. As Epac1 has been shown to play roles in cAMP-dependent Rap1 activation and cell adhesion in various cell types (38–39), our results suggest that PI3Kγ-mediated Rap1 activation is regulated separately from cAMP-mediated Rap1 activation by the use of different Rap-GEFs.

Our studies implicating two calcium and diacylglycerol activated Rap-GEFs in Rap-dependent integrin activation suggested a role for PLCγ in integrin α4β1 activation. PLCγ hydrolyzes 4,5 bisphosphate (PI(4,5)P2) into two second messengers, inositol 1,4,5 triphosphate (IP3) and diacylglycerol (DAG)(42–43). While IP3 releases calcium from intracellular stores, PLC generated DAG activates PKC and guanine exchange factors (GEFs) such as CalDAG-GEFI and CalDAG-GEFII (40–41). As PLCγ can be activated by PI3-Kinase (44), we tested PLCγ1 siRNA and pharmacological inhibitors and found that these reagents completely blocked Rap activation, integrin activation and myeloid cell adhesion.

Our studies clearly identified a role for the Rap effector RIAM in myeloid cell integrin activation, as RIAM knockdown suppressed integrin α4β1-mediated adhesion and ligand binding. Rap can also activate RAPL, another Rap effector protein found in lymphocytes. Rap1-GTP can bind to RIAM and promote talin localization to the leading edge of cells, since Rap1 is prenylated and thereby localized to the membrane.

In the studies presented here, we found that PI3Kγ-mediated Rap1a and integrin α4β1 activation depend on PLCγ, Rap-GEFs CalDAG-GEFI and II and RIAM. In vivo, tumor-derived chemoattractants promote the recruitment and infiltration of myeloid cells to the tumor microenvironment in PI3Kγ-Rap1a-integrin α4β1-dependent adhesion to the tumor endothelium. Blockade of either integrin α4β1 or Rap1a suppresses tumor inflammation, angiogenesis and tumor progression in murine models of cancer (9–10). These results suggest that antagonists of integrin α4β1 or Rap1 could offer clinical benefit for cancer patients. An antagonist of integrin α4β1, the humanized antibody natalizumab (Tysabri), has been developed for clinical applications and is currently in use for the treatment of multiple sclerosis and inflammatory bowel disorders (48). While this antagonist offers significant clinical benefit in these autoimmune diseases, it also elevates the risk to 0.2% for complications associated with immunosuppression, including progressive multifocal leukencephalopathy (PML), a neurotoxic JC virus infection of the brain (49–50). Natalizumab use in cancer therapy has been considered based on observations that α4β1 promotes angiogenesis and inflammation in pre-clinical models of solid tumors (9–10) and that it promotes the survival of B cell leukemias and other hematological cancer cells (51), but it is currently unclear if the potential benefits will out weight the potential of long-term health risks. Rap1a might serve as an alternative target for cancer therapy. The small GTPases Ras and Rap are post-translationally modifed by isoprenylation, the addition of farnesyl or geranylgeranyl lipids to C-terminal CAAX moieties on Ras and Rap, respectively (52). Farnesyltransferase and geranylgeranyltransferase inhibitors have been investigated for cancer and immune disease therapy, as small GTPases plays key roles in cell proliferation and invasion pathways (52). Interestingly, bisphosphonates, which are in clinical practice as inhibitors of bone resorption due to osteoporosis, are potent inhibitors of geranylgeranyl biosynthesis, and thus Rap1, and have proven effective in the therapy of multiple myeloma and breast carcinoma (52–54). As further details of the Rap1a-integrin α4β1 pathway are revealed, it is likely that new targets and therapies for the suppression of tumor inflammation will be developed.

## Methods

### Ethics

All animal studies were carried out in strict accordance with the recommendations in the Guide for the Care and Use of Laboratory Animals of the National Institutes of Health. All protocols were approved by the Institutional Committee on the Use and Care of Animals of the University of California, San Diego. Discomfort and injury to animals was limited to that which was unavoidable in the conduct of scientifically valuable research. Analgesics, anesthetics, and tranquilizing drugs were as determined by our veterinarians and according to the guidelines set down in the NIH Guide for the Care and Use of Laboratory Animals. All experiments utilized overdose of the inhalant isoflurane (followed by cervical dislocation) for euthanasia, as consistent with the recommendations of the Panel on Euthanasia of the American Veterinary Medical Association. Use of donated human peripheral blood cells from the San Diego Blood Bank was approved by the Institutional Review Board for human subjects research of the University of California, San Diego.

### Statistical Analysis

All error bars indicate standard error of the mean (SEM) of 3 replicates (in vitro studies) or 10 replicates (animal studies). All studies were performed a minimum of three times. Statistical significance was assessed with the Student *t* test for in vitro studies and ANOVA for in vivo studies using SigmaXL 6.02. A value of *P*<0.05 was considered significant and signified by an asterisk.

### Purification of Myeloid Cells

Myeloid cells were purified from murine bone marrow or human buffy coats obtained from the San Diego Blood Bank using anti-CD11b magnetic bead affinity chromatography according to manufacturer’s directions (Miltenyi Biotec). To assess the purity of the CD11b+ cell population, allophycocyanin-labelled anti-CD11b antibodies was added to cells, and flow cytometry was performed. Cell were >95% CD11b+.

### Adhesion Assays

1×10^5^ calcein-AM labelled murine CD11b+ cells were incubated on plastic plates coated with 5 µg/ml recombinant soluble VCAM-1 (R&D Systems) for 30 min at 37°C in basal media, culture media from Lewis lung carcinoma cells (TCM) or DMEM containing 200 ng/ml SDF1α, IL-1β, IL-6, TNFα or VEGF-A (R&D Systems) and 1 µM inhibitors directed against PI3Kγ (TG100-115), PLCγ (U73122), Rap1-selective geranylgeranyltransferase inhibitor (GGTI-2147, GGTI-298), PKC (Ro-32-0432, Go6976). U73122, GGTI-2147, R0-32-0432 and Go6976 were from Calbiochem. TG100-115 was from Sanofi-Aventis. After washing three times with warmed medium, adherent cells were quantified using a plate fluorimeter (GeniosPro, TECAN). CD11b+ myeloid cells from WT, Rap1−/−, p110γ−/−, α4Y991A mice as well as siRNA-transfected and RapV12 and p110γCAAX-transfected cells were serum-starved for 4 h, and then incubated for 20 min on 5 µg/ml rsVCAM-1. Adherent cells were automatically quantified using a plate fluorimeter (GeniosPro, TECAN).

### Ligand Binding Assay

1×10^6^ CD11b+ cells isolated from WT, p110γ−/−, or Rap1a−/− mice were incubated with 200 ng/ml IL-1β, SDF-1α, IL-6, TNFα, VEGF-A or medium together with 1 mg/ml mouse VCAM-1/humanFc fusion protein (R&D Systems) for 3 min. Cells were washed twice and incubated with donkey anti-human-FC-PE antibody (Jackson Immunoresearch) then analyzed by FACs Calibur. Mean fluorescence intensity of treated cells was compared to that of unstimulated cells (basal). These ligand-binding assays were performed on WT CD11b+ cells and cells transfected with GFP/RapV12, GFP/p110γCAAX or siRNAs and on CD11b+ cells that were treated with 1 µM GGTI-2147 for 30 min at 37°C.

### Human Integrin Activation Assay

Expression of integrin α4 on CD11b+ cells was determined by flow cytometry to detect PE-conjugated R1/2 (rat anti-murine α4, eBioscience) or HP2/1 (mouse anti-human α4, Biogen Idec). To quantify integrin activation, 2.5×10^6^ freshly isolated human myeloid cells/ml were incubated in culture medium containing 10 µg/ml normal human immunoglobulin (12000C, Caltag) for 45 min on ice. These cells were then incubated in 200 ng/ml SDF-1α or 1 mM Mn2+ in the presence or absence of 1 µM GTTI-2147 plus 2.5 µg HUTS21 (β1 activation epitope, BD-Bioscience), P4C10 (total β1, Chemicon), or IgG2 control for 10 min at 37°C, followed by Alexa 488 goat-anti-mouse antibodies for 20 min on ice. Bound antibody was quantified by flow cytometry.

### siRNA and Plasmid Transfections

Purified CD11b+ cells were transfected with 2 µg/1×10^6^ cells pcDNA 3.1p110γCAAX or pcDNA3.1 RapV12 or pGFPMax (Lonza) using AMAXA Mouse Macrophage Nucleofection Kits or with 100 nM of siRNA targeting integrin α4 (Mm_Itga4_1; Mm_Itga4_2), Rap1a (Mm_Rap1a_1; Mm_Rap1a_7), RIAM (Mm_Apbb1ip_1; Mm_Apbb1ip_3), CalDAG-GEFI (Mm_Rasgrp2_1; Mm_Rasgrp2_2), CalDAG-GEFII (Mm_Rasgrp1_1; Mm_Rasgrp1_2), Epac1 (Mm_Rapgef3_4; Mm_Rapgef3_5), Epac2 (Mm_Rasgrp4_3;Mm_Rasgrp4_5), PLCγ (Mm_Plcg1_1; Mm_Plcg1_2) or Non-silencing siRNA (Ctrl_AllStars_1), which were purchased from Qiagen. After transfection, cells were cultured for 24 h in culture media containing 20% serum. Each siRNA was tested individually for efficient knockdown of protein expression and for inhibition of adhesion or ligand binding. To control for off-target effects of siRNAs, we used siRNAs that had been previously validated to have no major off-target effect, and we used two or more distinct siRNAs per target.

### Gene Expression

Total RNA was isolated from primary myeloid cells using RNeasy Kit (Qiagen). cDNA was prepared from 500 ng RNA from each sample and qPCR was performed using primer sets for integrin α4 (*Itga4),* CalDAG-GEFI *(Rasgrp2;),* CalDAG-GEFII *(RasGrp1),* Epac1 *(Rapgef3),* Epac2 *(Rapgef4),* PLCγ *(Plcg1),* RIAM (*Apbb1ip*) from Qiagen (QuantiTect Primer Assay) or GAPDH (*Gapdh*) sense primers 5′CATGTTCCAGTATGACTCCACTC3′ and anti-sense primers 5′GGCCTCACCCCATTTGATGT′. Relative expression levels were normalized to *gapdh* expression according to the formula <2^∧^- (Ct *gene of interest* – Ct *gapdh*)>. Fold inhibition in expression levels were calculated by comparative Ct method <2^∧^- (ddCt)> (47).

### Rap1 Activation Assay

Rap activity was measured in BM cells after incubation for 30 min at 37°C in serum free media in the presence or absence of 1 µM TG100-115, followed by stimulation with basal medium or medium containing 200 ng/ml IL-1β, IL-6, SDF-1α (R&D Systems). GTP-Rap was purified using an RapGTPase pulldown assay kit (Thermo Scientific) from 1 mg cell lysate by addition of RalGDS-GST fusion proteins and glutathione-conjugated sepharose beads. GTP-Rap and total Rap were detected by immunoblotting with anti-Rap antibodies.

### Bone Marrow Transplantation

Bone marrow derived cells were aseptically harvested from 6–8 week-old female mice by flushing leg bones of euthanized mice with phosphate buffered saline containing 0.5% BSA and 2 mM EDTA, incubating cells in red cell lysis buffer (155 mM NH_4_Cl, 10 mM NaHCO_3_ and 0.1 mM EDTA) and centrifuging over Histopaque 1083. Approximately 5×10^7^ BMDC were purified by gradient centrifugation from the femurs and tibias of a single mouse. Two million cells were intravenously injected into tail veins of each lethally irradiated (1000 rad) 6-week old syngeneic recipient mouse. After 4 weeks of recovery, tumor cells were injected in C57BL/6 mice transplanted with BM from Rap1a−/− or WT.

### Tumor Studies

C57BL/6 LLC cells were obtained from the American Type Culture Collection (ATCC). Cells were cultured in antibiotic- and fungicide-free DMEM media containing 10% serum; cells tested negative for mycoplasma using the Mycoplasma Plus PCR primer set from Stratagene (La Jolla, CA). 5×10^5^ LLC cells were injected subcutaneously into syngeneic 6-to 8-week old wildtype, Rap1a−/− or BMT mice. Tumors dimensions were recorded at regular intervals. Tumors were excised at 21 days. Tumor weights were obtained, and tumors were cryopreserved in O.C.T., cryosectioned and immunostained for F4/80+ using BM8 (eBioscience) and for CD31 using MEC13.3 (BD Bioscience). Slides were counterstained with DAPI (Invitrogen). Tissues were analyzed using Metamorph (Version 6.3r5, Molecular Devices).

### Quantification of Myeloid Cells in Tissues by Flow Cytometry

To quantify myeloid cells in tissues, tumors were excised, minced and digested to single cell suspensions for 1 h at 37°C in 5 ml of Hanks Balanced Salt Solution (HBSS, GIBCO) containing 1 mg/ml Collagenase type IV, 10 µg/ml Hyaluronidase type V and 20 units/ml DNase type IV (Sigma). Red blood cells were solublized with RBC Lysis Buffer (eBioscience) and then cells were incubated in FC-blocking reagent (BD Bioscience), followed by anti-CD11b-APC (M1/70), anti-Gr1-FITC (RB6-8C5) and anti F4/80 (BM8) (eBioscience). To exclude dead cells, 0.5 µg/ml propidium iodide was added.

## Supporting Information

Figure S1 **Efficiency of integrin and Rap1a gene knockdown and inhibition in primary myeloid cells.** (A) Integrin α4 expression (dark line) in myeloid cells from WT and Rap1a−/− bone marrow cells was quantified by flow cytometry. IgG control staining is shown in grey. (B) Validation of siRNA mediated knockdown of Rap1a in myeloid cells by Western blotting. Actin levels were used as loading control. Non-silencing control was set to 1. (C) Percent adhesion of WT chemoattractant-treated myeloid cells to VCAM-1 exposed to increasing concentrations of the geranylgeranyltransferase inhibitor (GGTI-2147). (D) Surface expression of α4β1 in myeloid cells after transfection with integrin α4 (black) or non-silencing (gray) siRNA. Histogram was acquired by flow cytometry. (E) Relative integrin α4 mRNA levels in myeloid cells after siRNA-mediated gene knockdown. Error bars indicate S.E.M.
(TIF)

Figure S2 **Rap inhibitor blocks α4β1 ligand binding.** Mean fluorescence intensity (MFI) of VCAM-1/Fc bound to myeloid cells derived from WT -treated with medium or 10 µM geranylgeranyltransferase inhibitor (GGTI-2147), in the absence (basal) or presence of IL-1β and SDF-1α (n = 3). *P<0.01 vs basal.
(TIF)

Figure S3 **RapGEFs in myeloid cell adhesion**. (A) Relative mRNA expression levels of CalDAG-GEFI, CalDAG-GEFII, Epac1, and Epac2 in myeloid cells. (B) Left: Relative RapGEF mRNA levels in myeloid cells after siRNA mediated knockdown. Non silencing control was set to 1. (C) Relative mRNA levels of PLCγ in myeloid cells after transfection with PLCγ or control siRNA. Non-silencing control was set to 1 (n = 3). (D) Percent adhesion of chemoattractant-treated WT myeloid cells to VCAM-1 in the presence of increasing concentrations of the PLCγ inhibitor U73122.
(TIF)

Figure S4 **Myeloid cell integrin α4β1 activation is PKC independent but RIAM dependent.** **(**A) Adhesion of WT chemoattractant-treated myeloid cells to VCAM-1 in the presence of 1 µM panPKC inhibitor Ro-32-0432 (n = 3), *P<0.01 vs basal. (B) Adhesion of WT chemoattractant-treated myeloid cells and WT myeloid cells ectopically expressing active p110γ (p110γCAAX), active Rap (RapV12), or empty vector (control) in the absence (empty) or presence (filled) of 1 µM PKC-α/β inhibitor (Go6976) (n = 3), *P<0.01 vs basal. (C) Relative mRNA levels of RIAM in myeloid cells after transfection with RIAM or control siRNA. Non-silencing control was set to 1 (n = 3).
(TIF)

Figure S5 **Rap1a promotes myeloid cell trafficking during tumor inflammation, thereby supporting tumor growth.** (A-B) Representative experiment showing (A) tumor volume and (B) weight of LLC tumors grown over 21 days in WT and Rap1a−/− mice (n = 10). (C) Percentage of Gr1+CD11b+ and (D) F4/80+ tumor-infiltrating myeloid cells in WT and Rap1a−/− tumors, *P<0.01 vs WT.
(TIF)
