# Supplementary figures and images for: PI3-Kinase γ Promotes Rap1a-Mediated Activation of Myeloid Cell Integrin α4β1, Leading to Tumor Inflammation and Growth
Source: PLoS One. 2013 Apr 2;8(4):e60226. doi: 10.1371/journal.pone.0060226 (PMC3614555; doi:10.1371/journal.pone.0060226)

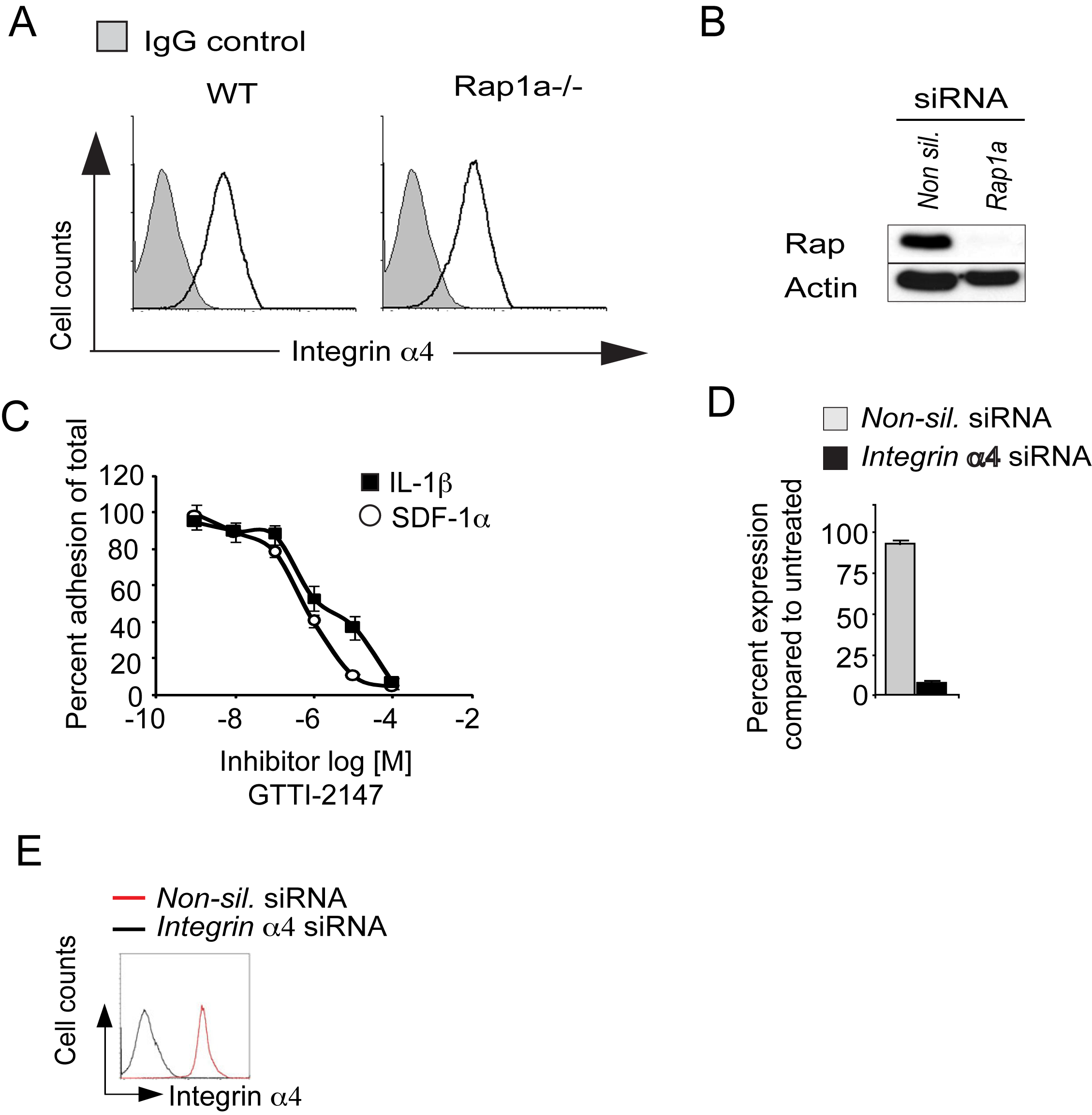

Supplement: Figure S1 — Efficiency of integrin and Rap1a gene knockdown and inhibition in primary myeloid cells. (A) Integrin α4 expression (dark line) in myeloid cells from WT and Rap1a−/− bone marrow cells was quantified by flow cytometry. IgG control staining is shown in grey. (B) Validation of siRNA mediated knockdown of Rap1a in myeloid cells by Western blotting. Actin levels were used as loading control. Non-silencing control was set to 1. (C) Percent adhesion of WT chemoattractant-treated myeloid cells to VCAM-1 exposed to increasing concentrations of the geranylgeranyltransferase inhibitor (GGTI-2147). (D) Surface expression of α4β1 in myeloid cells after transfection with integrin α4 (black) or non-silencing (gray) siRNA. Histogram was acquired by flow cytometry. (E) Relative integrin α4 mRNA levels in myeloid cells after siRNA-mediated gene knockdown. Error bars indicate S.E.M. (TIF) [file pone.0060226.s001.tif]

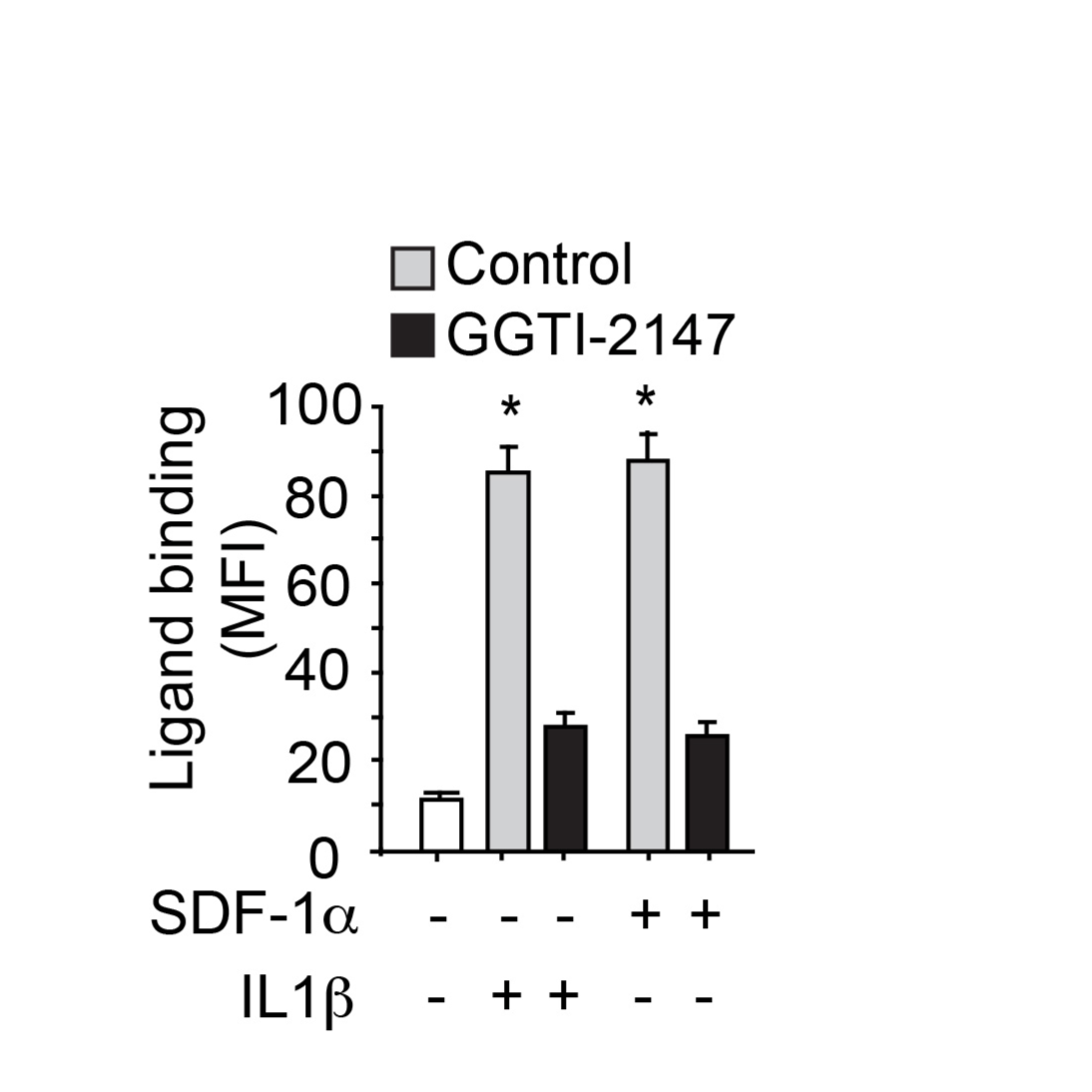

Supplement: Figure S2 — Rap inhibitor blocks α4β1 ligand binding. Mean fluorescence intensity (MFI) of VCAM-1/Fc bound to myeloid cells derived from WT -treated with medium or 10 µM geranylgeranyltransferase inhibitor (GGTI-2147), in the absence (basal) or presence of IL-1β and SDF-1α (n = 3). *P<0.01 vs basal. (TIF) [file pone.0060226.s002.tif]

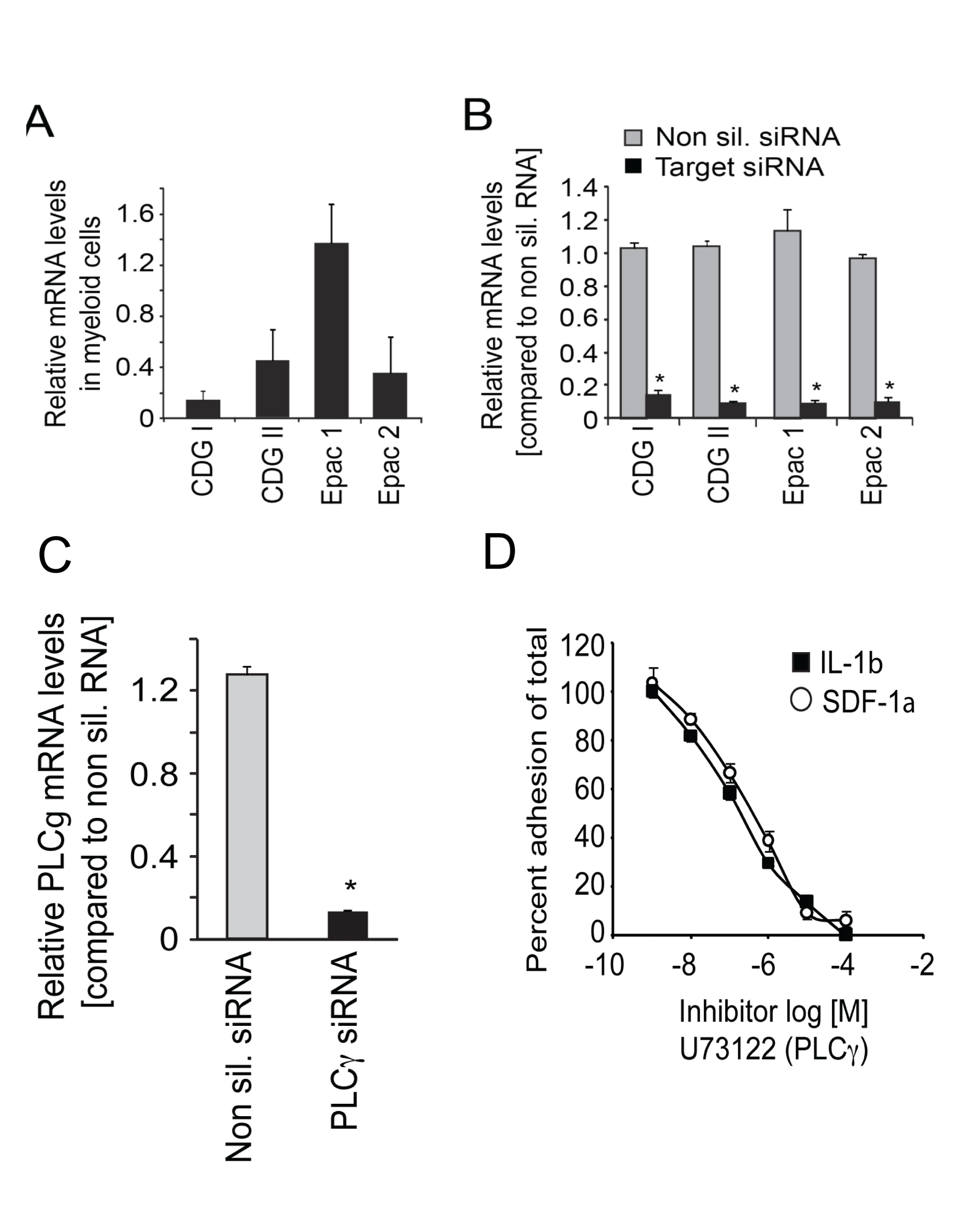

Supplement: Figure S3 — RapGEFs in myeloid cell adhesion. (A) Relative mRNA expression levels of CalDAG-GEFI, CalDAG-GEFII, Epac1, and Epac2 in myeloid cells. (B) Left: Relative RapGEF mRNA levels in myeloid cells after siRNA mediated knockdown. Non silencing control was set to 1. (C) Relative mRNA levels of PLCγ in myeloid cells after transfection with PLCγ or control siRNA. Non-silencing control was set to 1 (n = 3). (D) Percent adhesion of chemoattractant-treated WT myeloid cells to VCAM-1 in the presence of increasing concentrations of the PLCγ inhibitor U73122. (TIF) [file pone.0060226.s003.tif]

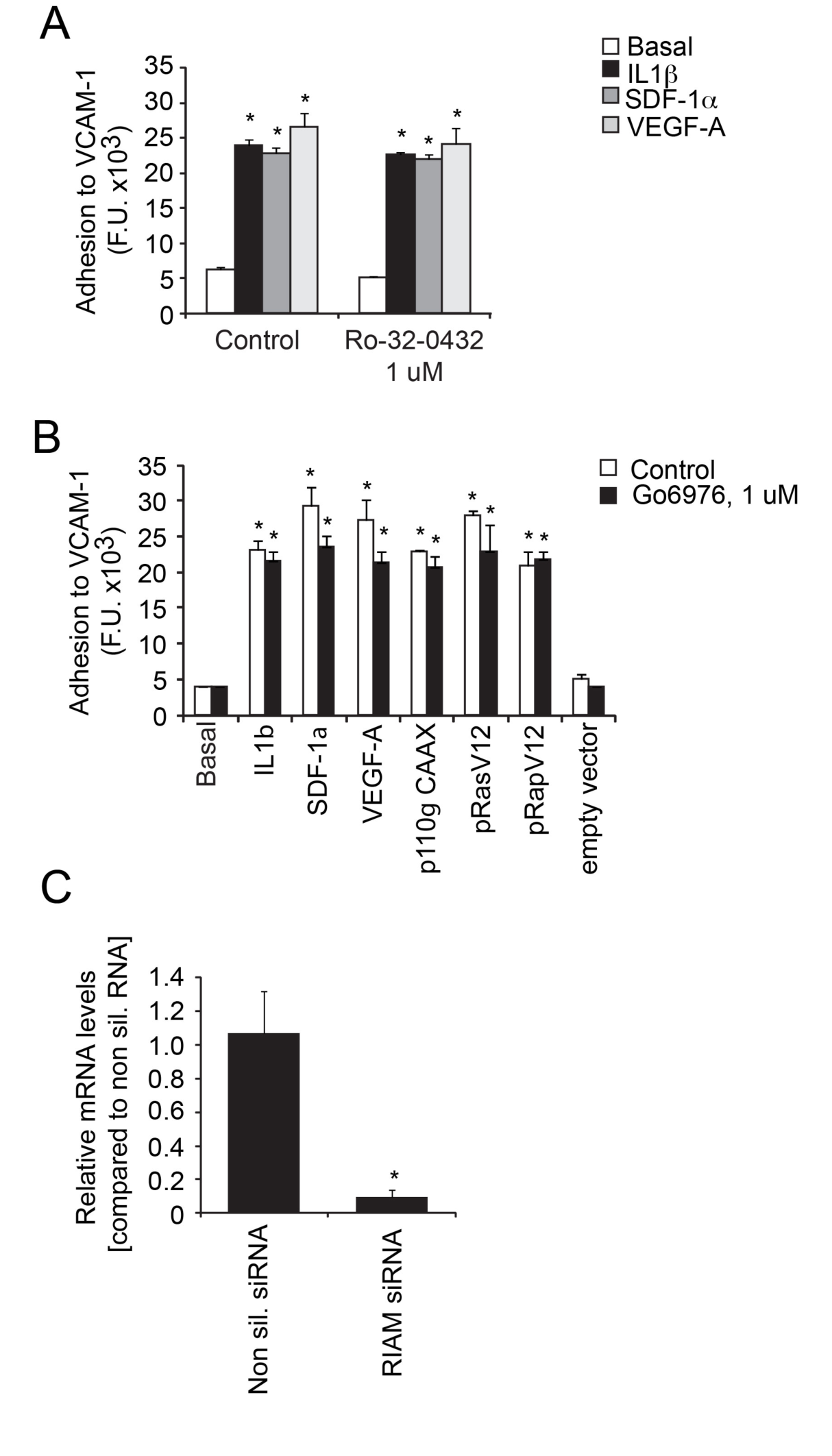

Supplement: Figure S4 — Myeloid cell integrin α4β1 activation is PKC independent but RIAM dependent. (A) Adhesion of WT chemoattractant-treated myeloid cells to VCAM-1 in the presence of 1 µM panPKC inhibitor Ro-32-0432 (n = 3), *P<0.01 vs basal. (B) Adhesion of WT chemoattractant-treated myeloid cells and WT myeloid cells ectopically expressing active p110γ (p110γCAAX), active Rap (RapV12), or empty vector (control) in the absence (empty) or presence (filled) of 1 µM PKC-α/β inhibitor (Go6976) (n = 3), *P<0.01 vs basal. (C) Relative mRNA levels of RIAM in myeloid cells after transfection with RIAM or control siRNA. Non-silencing control was set to 1 (n = 3). (TIF) [file pone.0060226.s004.tif]

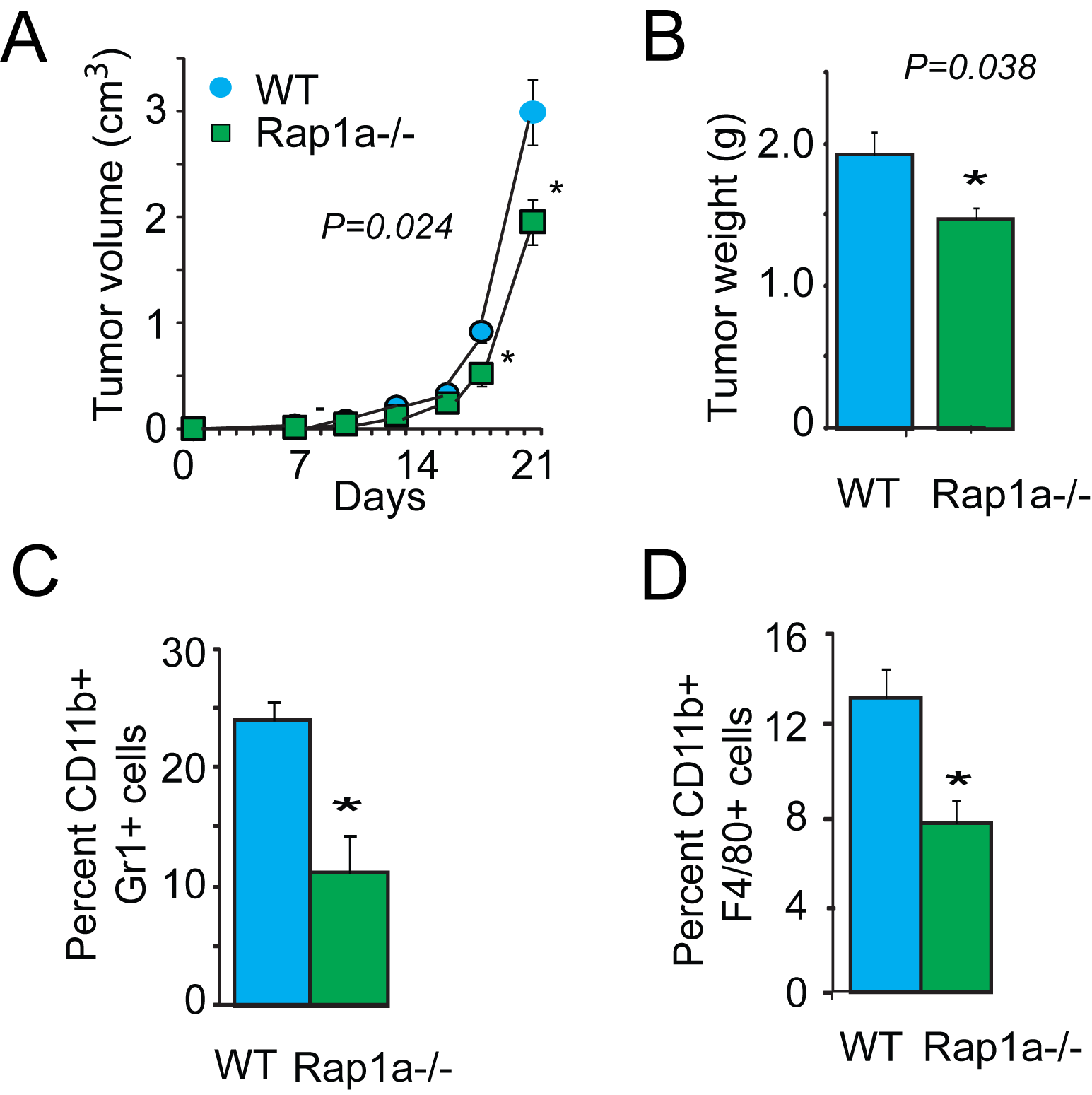

Supplement: Figure S5 — Rap1a promotes myeloid cell trafficking during tumor inflammation, thereby supporting tumor growth. (A-B) Representative experiment showing (A) tumor volume and (B) weight of LLC tumors grown over 21 days in WT and Rap1a−/− mice (n = 10). (C) Percentage of Gr1+CD11b+ and (D) F4/80+ tumor-infiltrating myeloid cells in WT and Rap1a−/− tumors, *P<0.01 vs WT. (TIF) [file pone.0060226.s005.tif]
